# Effects of Co Doping on the Growth and Photocatalytic Properties of ZnO Particles

**DOI:** 10.3390/molecules27030833

**Published:** 2022-01-27

**Authors:** Lanqin Tang, Yin Jia, Zhishang Zhu, Yue Hua, Jun Wu, Zhigang Zou, Yong Zhou

**Affiliations:** 1 College of Chemistry and Chemical Engineering, Yancheng Institute of Technology, 9 Yingbin Avenue, Yancheng 224051, China; xiaoyu2898@sina.com (Y.J.); DouglasM2000@163.com (Z.Z.); JoannaVu22@163.com (Y.H.); swujun@163.com (J.W.); 2 National Laboratory of Solid State Microstructures, Collaborative Innovation Center of Advanced Microstructures, School of Physics, Nanjing University, Nanjing 210093, China; zgzou@nju.edu.cn; 3 Eco-Materials and Renewable Energy Research Center (ERERC), Nanjing University, Nanjing 210093, China

**Keywords:** ZnO, Co-doped, flower-like, photocatalytic properties, chemical method

## Abstract

The present work reports on the synthesis of ZnO photocatalysts with different Co-doping levels via a facile one-step solution route. The structural and optical properties were characterized by powder X-ray diffraction (XRD), field emission scanning electron microscopy (FESEM), transmission electron microscopy (TEM), energy dispersive spectroscopy (EDS), and UV-Vis diffuse reflectance spectra. The morphology of Co-doped ZnO depends on the reaction temperature and the amount of Co and counter-ions in the solution. Changes with the c-axis lattice constant and room temperature redshift show the replacement of Zn with Co ions without changing the wurtzite structure. Photocatalytic activities of Co-doped ZnO on the evolution of H_2_ and the degradation of methylene blue (MB) reduce with the doping of Co ions. As the close ionic radii of Co and Zn, the reducing photocatalytic activity is not due to the physical defects but the formation of deep bandgap energy levels. Photocurrent response experiments further prove the formation of the recombination centers. Mechanistic insights into Co-ZnO formation and performance regulation are essential for their structural adaptation for application in catalysis, energy storage, etc.

## 1. Introduction

The photocatalytic transformation has received continuous attention with the depletion of fossil fuels and the intensification of environmental pollution [1]. Traditional semiconductors, such as TiO_2_, ZnO, etc., are usually applied as photocatalysts [2]. Controlling the morphologies of catalysts with specific exposed facets will result in efficient separation of the electron–hole pairs [3]. Doping ions is an effective way to reduce the bandgap of catalysts and improve the ability of visible-light harvesting [4,5]. Consequently, considerable interest has been focused on synthesizing photocatalysts with controllable morphologies and regulating their photocatalytic properties by a doping-based method.

Zinc oxide (ZnO) has been actively investigated due to its unique optical, electronic, and piezoelectric properties, such as solar cells [6], piezotronics [7], UV detectors [8], gas sensors [9], and light-emitting diodes (LEDs) [10]. Additionally, ZnO is a promising semiconductor with the richest range of morphologies and has been found to display good photoconductivity and high transparency in the visible region [11]. There are several methods available for controlling morphologies at present, such as the sputtering deposition technique [12], chemical vapor deposition [13], pulsed laser deposition [14], and the co-precipitation process [15]. Among these methods, the aqueous solution method appears more favorable in an economical and large-scale production [16,17,18]. Particles with specific crystal facets and complex corrugated particles with tips also have good photocatalytic activity and dispersion properties [19,20,21,22]. Based on previous studies, the microstructure, morphology, and luminescence performance of particles are extremely sensitive to the conditions of their preparation.

The Co-doping element is of much interest among the transition metal ions. Substituting the non-magnetic element Zn ion with the Co ion can introduce ferromagnetic behavior while retaining its semiconducting properties. There were also some reports on Co-doped ZnO nanoparticles, such as nano-crystalline powders [23], nanowires [24], etc. Different methods were applied to prepare Co-doped ZnO particles. For example, Chang et al. use the solvothermal method and calcination to dope Co^2+^/Co^3+^ ions [25]. A similar process conducted at 150 °C is also reported by Fang and Wang et al. [26]. Asif et al. reported bimetallic Co-Ni/ZnO cubes through the hydrothermal process [27]. It is suggested that structural, optical, and photocatalytic properties largely depended on the synthesis method. The aqueous solution method is more facile for regulating the structures of catalysts, which influence their properties. Furthermore, previous applications mainly focus on sensing, Raman, magnetism, and antibacterial activity. Therefore, it seems necessary to study the growth parameters and Co-dopant effects on the photocatalytic activity of the Co-doped ZnO.

A simple one-step aqueous solution route is reported for synthesizing Co-doped ZnO nanoparticles. No organic solvents or high-temperature treatments are used. The presented doping process has been achieved at an attractive low temperature (50 °C). Well-defined flower-like ZnO and Co-ZnO particles have been directly obtained. The present work investigates the formation process and photocatalytic activities of pure and cobalt-doped ZnO nanoparticles. It is worth noting that we used the Co-doped ZnO structure as a model system to understand the growth and doping-induced photocatalytic property mechanism. Effects of growth parameters such as doping content, reaction temperature, and counter-ions on the formation of particles are studied. The influences of Co-doping on structural, optical, morphological, and photocatalytic activities, including methylene blue degradation and H_2_ evolution, are also investigated in detail. Due to the similar ionic radii of Co and Zn, the replacement of Zn with Co creates deep bandgap levels and acts as the recombination centers, and eventually leads to the reduction of the photocatalytic activity. Therefore, doping species should be paid more attention. This method opens a new avenue for regulating the microstructure and properties of photocatalytic inorganic compounds.

## 2. Experimental

### 2.1. Materials

The starting chemical reagents used in this work were: zinc acetate dihydrate (Zn(CH_3_COO)_2_·2H_2_O), cobaltous chloride hexahydrate (CoCl_2_·6H_2_O), cobalt acetate tetrahydrate (Co(CH_3_COO)_2_·4H_2_O), and sodium hydroxide (NaOH), all of which were from Sinopharm Chemical Reagent Co., Ltd., Shanghai, China, and were used as analytic grade without further purification.

### 2.2. Experimental Procedure

In a typical synthesis, 50 mL of a mixed solution containing 0.05 mol of Zn(CH_3_COO)_2_·2H_2_O and a certain amount of 1.0 M CoCl_2_·6H_2_O solution were prepared in a three-necked flask under stirring. Then, 50 mL of 2.0 M NaOH solution was added dropwise to the flask at a rate of 1.0 mL/min at 50 °C, resulting in the formation of precipitates. After adding the above-mixed solution, the obtained precipitates were washed with distilled water and dried at 70 °C in the air for 24 h. The molar ratio of Co^2+^/Zn^2+^, R, was varied from 0.2:100 to 0.8:100. Experimental conditions for typical Co-doped ZnO samples are listed in Table 1.

### 2.3. Characterization of Materials

The crystalline structures were characterized using a Rigaku D/Max-RA Cu Kα diffractometer employing a scanning rate of 0.01° S^−1^ in the 2θ ranges from 30 to 70°. Zinc oxide crystallites were quantitatively characterized and compared with the calculated texture coefficient, *T*_c(hkl)_. The texture coefficient, *T*_c(hkl)_, is defined as follows [28]: *T*_c(hkl)_ = (*I*_(hkl)_/_Ir(hkl)_)/[1/*n*Σ(*I*_(hkl)_/*I*_r(hkl)_)], where *T*_c(hkl)_ is the texture coefficient, *n* is the number of peaks considered, *I*_(hkl)_ are the intensities of the peaks of obtained zinc oxide samples, and *I*_r(hkl)_ is the peak intensities indicated in the JCPDS 36-1451 corresponding to the randomly oriented crystallites. The lattice parameter, c, of the samples is calculated using the formula: sin^2^*θ* = *λ*^2^/4[4(*h*^2^ + *hk* + *k*^2^)/3*a*^2^ + *l*^2^/*c*^2^], where *θ* is the diffraction angle, *λ* is the incident wavelength, and *h*, *k,* and *l* are all Miller’s indices. The morphologies of the samples were studied by field emission scanning electron microscope (FESEM, JEOL JSM-6700F, Tokyo, Japan) and a transmission electron microscope (TEM, JEOL-1230, Tokyo, Japan). Energy dispersive spectroscopy (EDS) measurements were applied to determine the dopant content of cobalt ions in the Co-ZnO particles. The response curves were measured with the light on/off cycles at 0 V versus SEC (saturated calomel electrode).

### 2.4. Measurement of Photocatalytic Activity

Methylene Blue (MB) dye was used to evaluate the photocatalytic activity of doped and pure ZnO particles in response to UV light. A 6 W UV lamp with a wavelength of 365 nm was employed as the light source. Then, 50 mg of photocatalysts was dispersed in 100 mL of 10 mg/L of the MB aqueous solution in a 100 mL beaker. The suspension was stirred in the dark for 30 min to ensure the adsorption and desorption equilibrium of MB on the particle surface. Subsequently, the suspension was irradiated with simulated UV light. The distance between the light source and the surface of the solution was 6 cm. After every 30 min of irradiation, 5 mL of the suspension was extracted and then centrifuged to separate particles from the supernatant. The obtained solution was analyzed by recording variations in the absorption band (663 nm) in the UV-Vis spectra of MB using a UV-2100 spectrophotometer.

Photocatalytic activity in H_2_ production was examined using a 100 mL Pyrex flask. The flask was sealed with a silicone rubber septum. A 300 W Xe arc lamp was used as the light source. In a typical experiment, 50.0 mg of photocatalyst was mixed in 100 mL of an aqueous solution containing 0.25 M NaS_2_ and 0.25 M NaSO_3_. Before irradiation, the suspension was bubbled with nitrogen for 30 min to remove any dissolved oxygen and ensure the system was under anaerobic conditions. Magnetic stirring was used throughout the reaction to keep the photocatalyst particles in a suspension state. In addition, 0.4 mL of gas was sampled intermittently through the septum and analyzed using a gas chromatograph equipped with a thermal conductivity detector (TCD).

## 3. Results and Discussion

### 3.1. Structural and Morphological Characteristics

**Figure 1:**
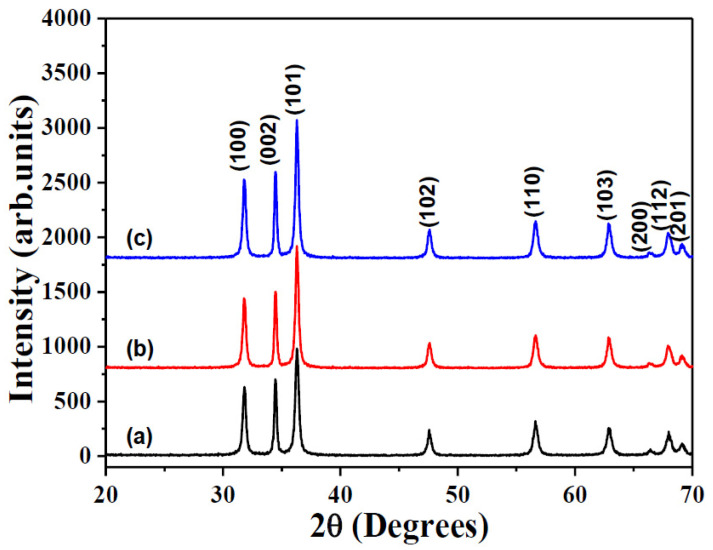
The powder XRD patterns of pure ZnO sample-S_0_ (**a**), sample-S0.2 (**b**), and sample-S0.8 (**c**) photocatalysts.

Cobalt was selected for its expected ease in doping due to the similar ionic radius (0.58 Å) to Zn (0.60 Å). Figure 1 displays XRD patterns of pure ZnO powder (sample-S_0_), Co-doped sample-S0.2, and sample-S0.8 photocatalysts. In each case, the characteristic (100), (002), (101), (102), (110), (103), (200), (112), and (201) reflections of simple wurtzite phase (ZnO, JCPDS No.36-1451) were observed, and no XRD patterns arising from other phases appeared. The XRD patterns of Co-doped ZnO samples show the same characteristics as undoped ZnO, consisting of only peaks corresponding to ZnO. This is because XRD has a relatively poor detection limit of around 1% by volume. The sharp and intense peaks in these structures indicate that all samples are highly crystallized. There is a reduction of the c-axis lattice constants with the increase of Co-dopant level, to 5.2091 Å (sample-S_0_), 5.2086 Å (sample-S0.2), and 5.2079 Å (sample-S0.8). Since the difference in radii of Co and Zn is minimal, the changes in cell parameters with cobalt substitution in the lattice are rather small [28]. Therefore, the difference of lattice parameters could be attributed to Co incorporation, which indicates defect evolution in the lattice.

The texture coefficient values of these samples produced in the presence or the absence of Co were also calculated according to their three main corresponding X-ray diffraction peaks (Table 2). The (002) orientation with a high texture coefficient, *T*_c(002)_, is stronger than the (100) orientation and the (101) orientation. *T*_c(hkl)_ of 1 presents a sample with randomly oriented crystallites, where a larger value indicates an abundance of crystallites oriented to the (*hkl*) plane [29]. These results confirmed that the addition of Co did not change the (002) growth direction. Furthermore, with the loading of Co, the average crystal size of these samples decreased from 28.78 nm (sample-S_0_) to 14.39 nm (sample-S0.2) and 5.08 nm (sample-S0.8). Co led to a smaller crystal size of the produced zinc oxide particles.

**Figure 2:**
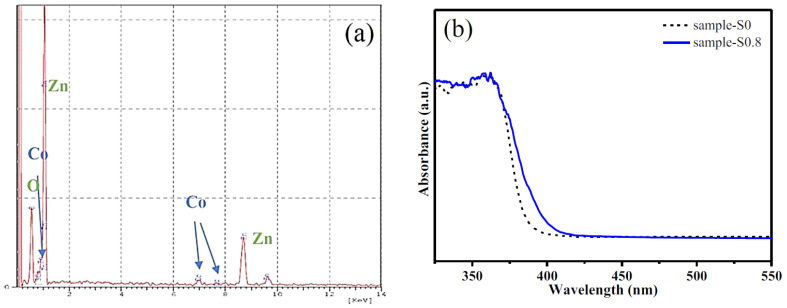
EDS (**a**) and UV (**b**) spectra of sample-S0.8.

The energy-dispersive spectra (EDS) further revealed that Co, Zn, and O exist in Co-based samples (Figure 2). Quantitative analysis of the atomic concentration (atom%) is listed in Supplementary Table S1. It can be found that the Co content in the samples increased from 0.12 to 0.38 atom%, based on the Co-doping levels. However, less Co was located in Co-doped ZnO crystallites than that provided in the precursor solution, indicating that some Co ions remain in the parent solution and do not become incorporated into the crystals. Furthermore, the band edge of the Co-doped ZnO sample-S0.8 shifted to the lower energy side, compared to the pure ZnO sample-S_0_ (Figure 2). The redshift phenomenon is mainly due to the sp–d exchange interactions between the band electrons and the localized d electrons of the Co^2+^ cations. These results confirmed that the addition of Co did not change the ZnO structure.

### 3.2. Morphological Characteristics

**Figure 3:**
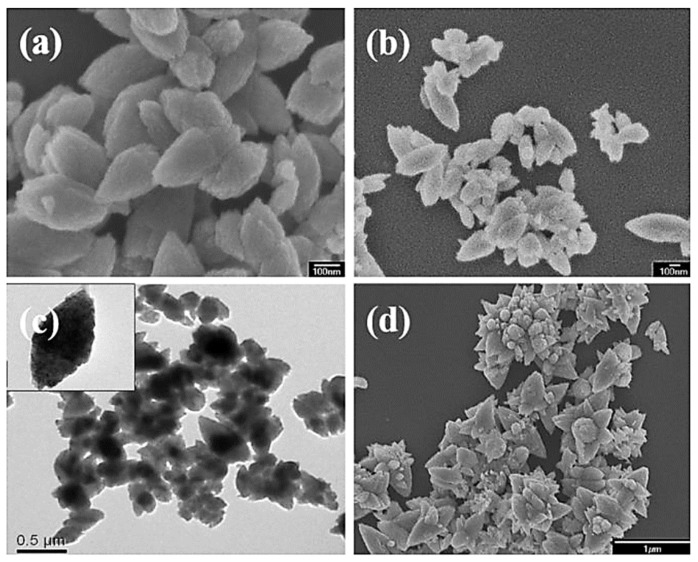
FESEM and TEM images of pure ZnO sample-S0 (**a**), and Co-doped sample-S0.2 (**b**,**c**) and sample-S0.8 (**d**).

From Figure 3a, it is apparent that pure ZnO exhibits an olive-like morphology with an average size of about 400 nm. With the addition of Co, the obtained sample-S0.2 also showed an olive-like morphology with a length of about 500 nm (Figure 3b,c). On the other hand, the morphology of sample-S0.8 produced after Co-doping changed significantly, and was mostly flower-like nanoclusters with an average size of 500 nm, which are comprised of much smaller nanorods (Figure 3d). Flower-like particles with enormous interface areas demonstrated good light-harvesting ability, and quickly transformed the light-generated charge in the photocatalytic processes, and had better catalytic ability than nanorods and nanoparticles [30]. These differences can be explained in terms of a thermodynamic barrier arising from the dopant (Co^2+^) that slows the nucleation and inhibits further growth of ZnO around a doped nanocrystal.

### 3.3. Mechanism

#### 3.3.1. Effects of Reaction Temperature on the Morphology of Co-Doped ZnO Particles

**Figure 4:**
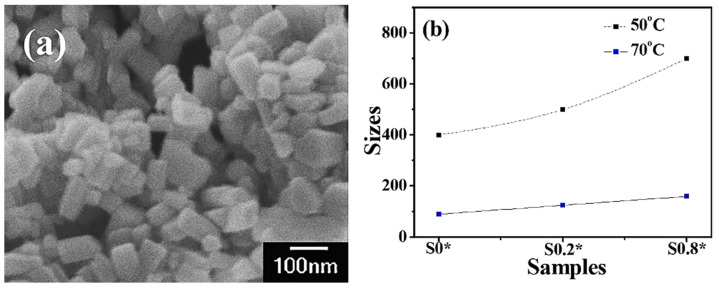
FESEM image of pure ZnO ((**a**) sample-S0*), and (**b**) the size distributions of sample-S0.2* and sample-S0.8*.

FESEM images of pure ZnO (sample-S_0_*), sample-S0.2*, and sample-S0.8* are shown in Figure 4. When the reaction temperature was increased to 70 °C, the obtained ZnO particles of sample-S_0_* with a size of about 90 nm were obtained, which is smaller than sample-S_0_ obtained at 50 °C (400 nm, Figure 4a). Higher temperature accelerates the growth process and allows the synthesis of small ZnO particles [31]. With the addition of Co, the obtained sample-S0.2* and sample-S0.8* had a larger size of about 150 nm, but a similar 1D rod-like morphology to sample-S_0_* (Figure 4b). This is quite different from particles obtained at the reaction temperature of 50 °C when 3D particles are formed. Higher temperature accelerates the growth process but does not change the growth characteristics of Co-doped ZnO samples. The morphology of Co-doped ZnO particles seems more dependent on reaction temperature than the doped amount of Co.

#### 3.3.2. Effects of Cobalt Counter-Ions on the Morphology of Co-Doped ZnO Particles

**Figure 5:**
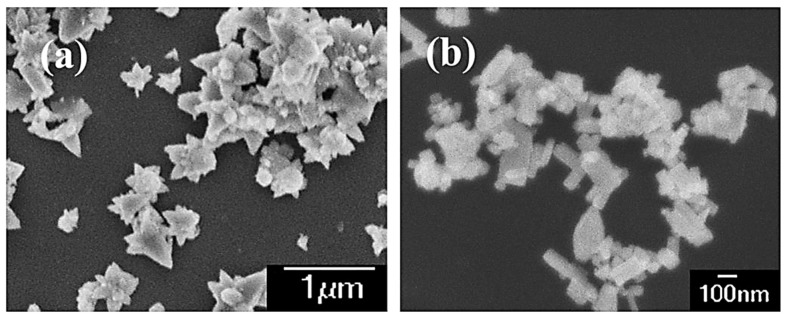
FESEM images of sample-C0.8#50 (**a**) and sample-C0.8#70 (**b**).

Cobalt acetate tetrahydrate (Co(CH_3_COO)_2_·4H_2_O, 0.2 mol% Co:ZnO) was also used as the Co source, and sample-C50 and sample-C70 were obtained when the reaction temperature was 50 and 70 °C, respectively. Figure 5a shows that when cobalt acetate tetrahydrate was used as the Co source instead of cobaltous chloride hexahydrate, flower-like particles composed of olives were produced (sample-C50, 50 °C). Interestingly, the flower-like particles of about 800 nm were larger than in sample-S0.8, which was obtained under nearly the same conditions, except for the amount of Co (0.8 mol% Co:ZnO). When the reaction was increased to 70 °C, rod-like particles similar to sample-S0.8* were obtained (sample-C70, Figure 5b). These results show that the cobalt counter-ion mainly affects the size of Co-doped ZnO particles.

#### 3.3.3. Photocatalytic Activity

**Figure 6:**
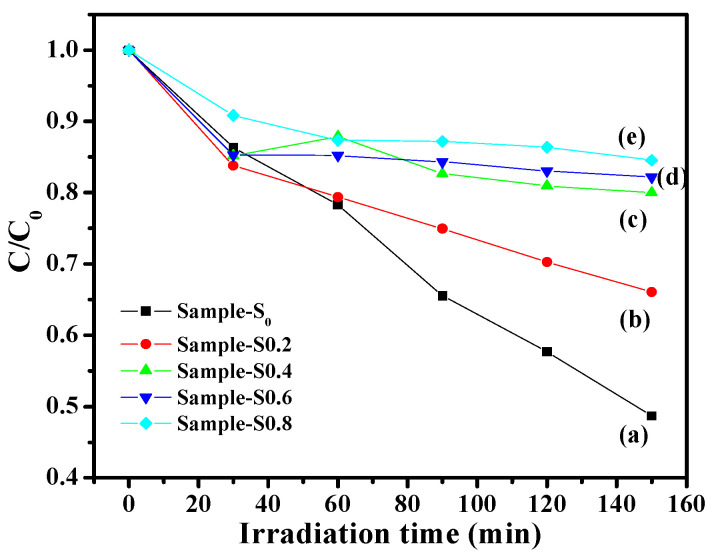
Extent of decomposition of the MB dye with respect to time intervals over ZnO (sample-S_0_ (**a**), and Co-doped ZnO photocatalysts: sample-S0.2 (**b**), sample-S0.4 (**c**), sample-S0.6 (**d**), and sample-S0.8 (**e**), under UV irradiation.

The photochemical reactivity of ZnO-based composites could be used for oxidative degradation of a variety of emerging contaminants, such as pharmaceuticals associated with toxicological impacts to aquatic environments [32]. The photochemical reactivity of Co-doped ZnO catalysts synthesized with different molar ratios of Co^2+^ to Zn^2+^ for degradation of MB has been performed under UV light irradiation at room temperature. Figure 6a shows that blank ZnO enables a near 52% degradation rate of 1 mg of MB with 150 min of UV light irradiation. With the addition of Co (0.2 mol% Co:ZnO), the photocatalytic activity of the obtained sample-S0.2 was lower than the blank, and about 34% of the 1 mg of MB was degraded (Figure 6b). Further increasing the amount of Co, the photocatalytic degradation rate of the obtained samples gradually decreased from 20% (sample-S0.4, Figure 6c) to 18% (sample-S0.6, Figure 6d), and eventually to 16% (sample-S0.8, Figure 6e). In our previous work, samples with different morphologies displayed a large difference in activities, and flower-like ZnO showed the best photodegradation rates [11]. However, flower-like Co-doped ZnO composites had the worst photodegradation rates in this work, which is probably because of the addition of Co. The kinetic model is discussed here to better understand the photocatalytic activity of synthesized photocatalysts. In general, the kinetics of photocatalytic degradation of organic pollutants on the semiconducting oxide has been established and can be well-described by the apparent first-order reaction, ln(C_0_/C_t_) = *k_app_t*, where *k_app_* is the apparent rate constant, C_0_ is the concentration of dyes after darkness adsorption for 30 min, and C_t_ is the concentration of dyes at time *t*. Supplementary Figure S1 shows the relationship between illumination time and the degradation rate of dyes for UV illumination. The linear correlation of the plots of ln(C_0_/C_t_) versus time suggests a pseudo-first-order reaction for MB dye.

The photochemical reactivity of sample-S_0_, sample-S0.2, sample-S_0_*, and sample-S0.2* was performed under UV light irradiation at room temperature. From Supplementary Figure S2, it is obvious that blank ZnO (sample-S_0_) obtained at 50 °C had better photocatalytic activity than that produced at 70 °C (sample-S_0_*). With the addition of Co (0.2 mol% Co:ZnO), the photocatalytic activity of the obtained sample-S0.2 and sample-S0.2* was lower than their corresponding blanks. Furthermore, sample-S0.2* showed a worse photocatalytic activity, and about 25% of 1 mg of MB was degraded under UV light irradiation for 150 min.

Sample-S0.2 was taken as a typical sample to study the degradation process. Supplementary Figure S3 shows the variances in the absorbance of MB with sample-S0.2 as the photocatalyst. The prominent absorption peak centered at 663 nm, and no blue- or red-shifts were observed during the photocatalytic procedure, implying that the decolorating process for MB is not ascribed to the N-demethylation [33]. Furthermore, after irradiation for 150 min, the discoloration of MB aqueous solution was apparent (see the inset in Supplementary Figure S2).

**Figure 7:**
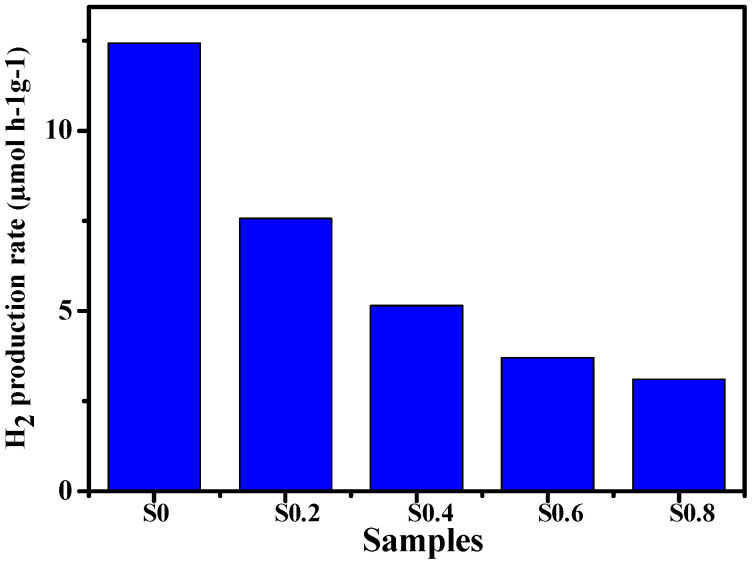
H_2_ evolution over ZnO (sample-S_0_), and Co-doped ZnO photocatalysts: sample-S0.2, sample-S0.4, sample-S0.6, and sample-S0.8.

We also evaluated these samples’ photocatalytic hydrogen evolution activities (Figure 7). Pure ZnO exhibited the best H_2_ production ability of about 50 μmol g^−1^ for 4 h (sample-S_0_). Furthermore, the H_2_ production rate also depends on the amount of Co. After doping with different mole fractions of Co, the photocatalytic H_2_ production rate significantly decreased. The lowest H_2_ production rate reached around 5.0 μmol h^−1^ g^−1^ over sample-S0.8, much lower than that over sample-S_0_. The results suggest that doping Co into ZnO is not practical for enhancing the H_2_ production rate.

**Figure 8:**
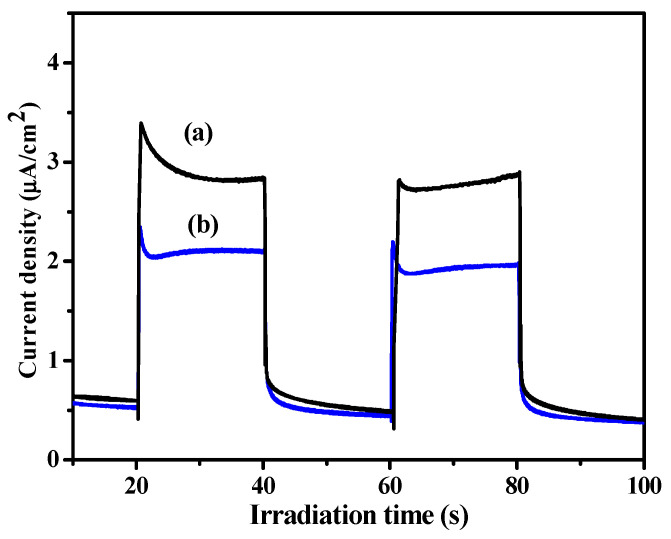
Photocurrent responses of ZnO (**a**) and Co-doped sample-S0.8 (**b**).

It is known that illumination of the semiconductor material with a photon flux of energy greater than the bandgap width causes absorption of photons and generation of additional electron–hole pairs. As a result of the generation, an increase in the concentration of the excess electrons in the conduction band and the extra holes in the valence band occurs. The photocatalytic activity depends not only on intrinsic crystallographic structures of photocatalysts but also on the separation of the electron–hole pairs [34]. The transient photocurrent response of the prepared samples was measured to examine the charge separation and migration properties. In this way, the separation of electron–hole pairs generated by light absorption gives rise to a photocurrent. The photo-response curves were measured with light on/off cycles at 0 V versus SEC (saturated calomel electrode). The pure ZnO exhibited a fast and uniform photocurrent response for each light-on and light-off process, and the photocurrent density of the ZnO electrode was detected as 3.3 μA/cm^2^ (Figure 8a). However, the photocurrent density dramatically decreased by about 50% after loading Co to ZnO (Figure 8b). The observed decrease in photocurrent intensity could be ascribed to combining the photo-generated electron–hole pairs with defects’ formation by loading Co to ZnO. The ionic radii of Co and Zn are close to each other. The mechanism of reducing photocatalytic activity by Co-doping is therefore not considered to be by creating physical defects. The efficient recombination centers for photo-generated excitons would be the deep bandgap energy levels between the valence and conduction bands [35]. The results well-parallel the photocatalytic performances for dye degradation and H_2_ production.

## 4. Conclusions

Co-doped ZnO particles directly from aqueous solutions at a low temperature (50 °C) have been successfully synthesized via a simple one-step solution route. The effects of Co-doping, reaction temperature, and Co counter-ions on the structural, morphological, and photocatalytic properties of doped ZnO particles were investigated. XRD spectra indicated that cobalt ions, in the oxidation state of Co2*p*, substitute Zn2*p* ions in the ZnO lattice without changing its wurtzite structure. According to the EDS spectra, the dopant content varied from 0.12% to 0.38%, depending on Co-doping levels. The reaction temperature and doped amount of Co significantly affected the morphology of doped ZnO particles. Pure ZnO exhibited an olive-like morphology, and the Co-ZnO particles took on the shape of olive-like and eventually flower-like nanoclusters depending on the amount of Co. The optical property of doped ZnO particles is determined by H_2_ evolution and the degradation of MB. The photocurrent intensity test proved the formation of recombination centers, leading to the reduction of photocatalytic activity of the Co-doping samples. This work showed that doping is a double-edged sword, and attention should be paid not only to the doping itself but also the doping species. Further work can focus on the study of serial doping metal ions and the regulations of the structural and catalytic properties.

## Figures and Tables

**Table 1:** Preparation parameters of various Co-doped ZnO samples.

Samples	Morphology	Zn^2+^ (mL)	Co^2+^ (mL)	Co/Zn (Theoretical Zn, mol %)	Reaction Temperature (°C)
S_0_	OLP	50	0.00	0	50
S_0.2_	OLP	50	0.10	0.2:100	50
S_0.4_	OLP	50	0.20	0.4:100	50
S_0.6_	FLP	50	0.30	0.6:100	50
S_0.8_	FLP	50	0.40	0.8:100	50
S_0_*	NR	50	0.00	0	70
S_0.2_*	NR	50	0.10	0.2:100	70
S_0.8_*	NR	50	0.40	0.8:100	70

OLP, NR, and FLP are abbreviations describing product morphology and refer to olive-like particles, nanorods, and flower-like particles, respectively.

**Table 2:** Texture coefficients of the obtained ZnO samples with different amounts of Co.

Samples	M_Co_^2+^:M_Zn_^2+^	Texture Coefficient		
		*T*_c(100)_	*T*_c(002)_	*T*_c(101)_
S_0_	0:100	0.91	1.29	0.80
S_0.2_	0.2:100	0.87	1.24	0.89
S_0.6_	0.6:100	0.88	1.24	0.88

## Data Availability

The data presented in this study are available in Supplementary Materials.

## Supplementary Materials

The following supporting information can be downloaded, Figure S1: Apparent rate constants of MB dye in the presence of ZnO (sample-S0, a) and Co-doped ZnO photocatalysts: sample-S0.2 (b), sample-S0.4 (c), sample-S0.6 (d), and sample-S0.8 (e) under UV irradiation.; Figure S2: Extent of decomposition of the MB dye with respect to time intervals over pure ZnO sample-S0 and sample-S0* (a,c), and Co-dopped ZnO photocatalysts: sample-S0.2 (b), and sample-S0.2* (d) under UV irradiation.; Figure S3: The absorbance of MB with sample-S0.8 as a function of irradiation time. Table S1: The composition of ZnO nanocrystals dependence on Co-doping levels.

## Sample Availability

Samples of the compounds are not available from the authors.
