# Supplementary material for: Effects of Co Doping on the Growth and Photocatalytic Properties of ZnO Particles
Source: Molecules. 2022 Jan 27;27(3):833. doi: 10.3390/molecules27030833 (PMC8840763; doi:10.3390/molecules27030833)
Supplement: Supplementary file 1 [file molecules-27-00833-s001.zip › molecules-1575017-supplementary.pdf]

Supplementary Materials

Effects of Co Doping on the Growth and Photocatalytic Properties of ZnO Particles

Lanqin Tang [1,2,3*], Yin Jia[1], Zhishang Zhu[1], Yue Hua[1], Jun Wu[1], Zhigang Zou[2, 3], Yong Zhou[2, 3*]

[1] College of Chemistry and Chemical Engineering, Yancheng Institute of Technology, 9 Yingbin Avenue, Yancheng, 224051, P. R. China

[2] School of Physics, National Laboratory of Solid State Microstructures, Collaborative Innovation Center of Advanced Microstructures, Nanjing University, Nanjing 210093, P. R. China

[3] Eco-Materials and Renewable Energy Research Center (ERERC), Nanjing University, Nanjing 210093, China

Corresponding author. E-mail: lanqin_tang@163.com; zhouyong1999@nju.edu.cn

Table S1. The composition of ZnO nanocrystals dependence on Co-doping levels.

Samples	Composition (atom %)		
	Zn	O	Co
S0.2	49.83	50.05	0.12
S0.4	49.70	50.12	0.18
S0.6	49.56	50.19	0.25
S0.8	49.46	50.16	0.38

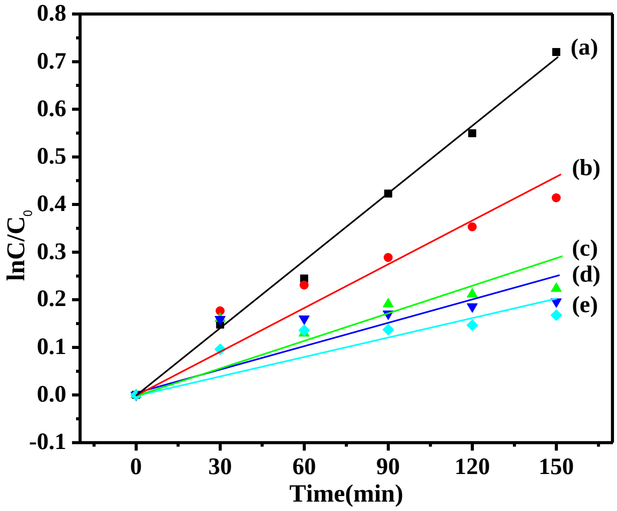

Figure S1. Apparent rate constants of MB dye in the presence of ZnO (sample-S0, a) and Co-doped ZnO photocatalysts: sample-S0.2 (b), sample-S0.4 (c), sample-S0.6 (d), and sample-S0.8 (e) under UV irradiation.

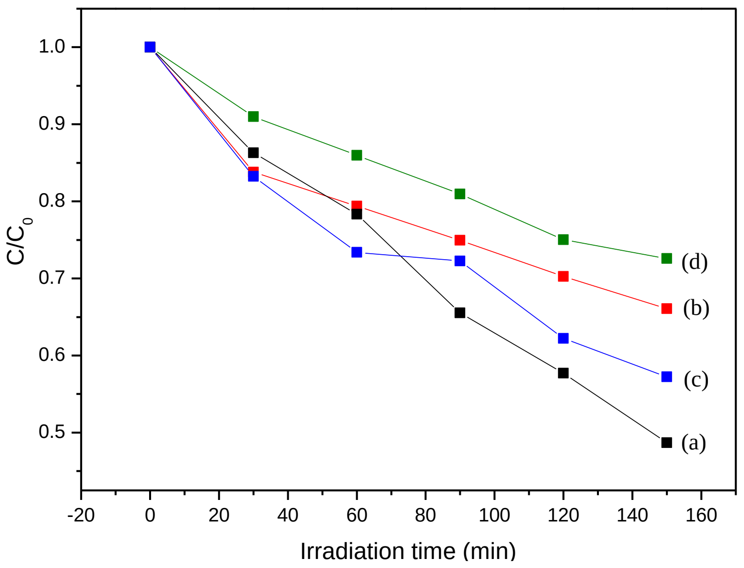

Figure S2. Extent of decomposition of the MB dye with respect to time intervals over pure ZnO sample-S0 and sample-S0* (a, c), and Co-dopped ZnO photocatalysts: sample-S0.2 (b), and sample-S0.2*(d) under UV irradiation.

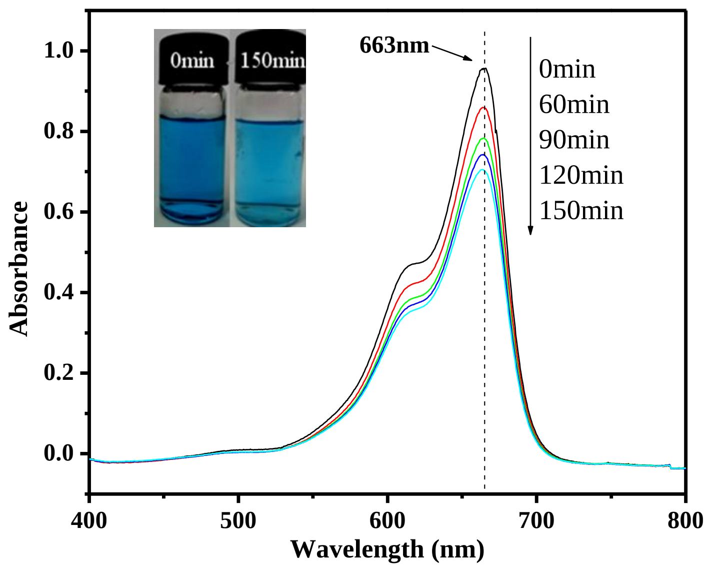

Figure S3. The absorbance of MB with sample-S0.8 as a function of irradiation time.
